# Predictive models for overall survival in breast cancer patients with a second primary malignancy: a real-world study in Shanghai, China

**DOI:** 10.1186/s12905-022-02079-0

**Published:** 2022-12-06

**Authors:** Ling Yuan, Yichen Chen, Xiaopan Li, Hua Jin, Jianwei Shi

**Affiliations:** 1 School of Public Health, Shanghai Jiaotong University School of Medicine, Shanghai, 200025 China; 2 Center for Disease Control and Prevention, Pudong New Area, Shanghai, 200136 China; 3 Fudan University Pudong Institute of Preventive Medicine, Pudong New Area, Shanghai, China; 4 Department of Health Management Center, Zhongshan Hospital, Shanghai Medical College of Fudan University, 180 Fenglin RD, Shanghai, 200032 China; 5 Department of General Practice, Yangpu Hospital, School of Medicine, Tongji University, 450 Tengyue RD, Shanghai, 200090 China; 6 Shanghai General Practice and Community Health Development Research Center, Shanghai, 200090 China; 7 Department of Social Medicine and Health Management, School of Public Health, Shanghai Jiaotong Universtiy School of Medicine, 227 South Chongqing RD, Shanghai, 200025 China

**Keywords:** Breast cancer, Second primary malignancy, Survival, Latency, Nomogram

## Abstract

**Background:**

The incidents of second primary malignancy (SPM) is increasing among breast cancer (BC) patients with long-term progression, adversely affecting survival. The purpose of this study was to screen independent overall survival (OS) risk factors and establish nomograms to predict the survival probabilities of BC patients with SPM.

**Method:**

A total of 163 BC patients with SPM were recruited during 2002–2015 from a total of 50 hospitals in Shanghai, China. Two nomograms to predict survival from primary BC and SPM diagnosis were constructed based on independent factors screened from multivariable analysis. The calibration and discrimination of nomograms were calculated in the training and validation cohorts.

**Results:**

The overall survival rates of BC patients with SPM were 88.34%, 64.42% and 54.66% at 5, 10 and 15 years, respectively. Factors of late TNM stage of SPM (HR = 4.68, 95% CI 2.14–10.25), surgery for SPM (HR = 0.60, 95% CI 0.36–1.00), SPM in the colon and rectum (HR = 0.49, 95% CI 0.25–0.98) and thyroid (HR = 0.08, 95% CI 0.01–0.61) independently affected the OS of BC patients with SPM (*p* < 0.05). In addition, a longer latency (≥ 5 years) was associated with better OS from BC diagnosis (*p* < 0.001). Older age (≥ 56) was associated with poor OS from SPM diagnosis (*p* = 0.019). Two nomograms established based on the above factors had better calibration and discrimination.

**Conclusion:**

The TNM stage of SPM, surgery for SPM, SPM sites, latency and age at BC diagnosis are independent factors for survival and the two nomograms may provide more personalized management for BC patients with SPM.

**Supplementary Information:**

The online version contains supplementary material available at 10.1186/s12905-022-02079-0.

## Introduction

Breast cancer (BC) is one of the most common cancers worldwide, accounting for nearly a quarter of the newly diagnosed cancer cases in women each year [1, 2]. Owing to the increasing awareness and use of mammography for earlier detection and advanced treatments, such as chemo- and radiotherapy, hormone therapy, and targeted drugs [3], the age-standardized 5-years net survival of female BC patients increased from 2000 to 2014 in most nations but varied from 66.1% in India to 90.2% in the United States [4]. In Southeast Asia, female BC patients in Japan and Korea had higher 5-years survival rates (above 86%), while China had a rate of 83.2%, which was higher than some other developing countries [4].

However, the second primary malignancy (SPM) that occurs after BC affects the long-term progression of BC patients [5]. SPM is an in situ cancer that develops after the first primary malignancy and does not metastasize or recur [6]. Due to factors such as side effects of cancer treatment, genetic mutations and environmental pollution, the incidence of SPM is rising [7]. A previous study revealed that SPM is one of the leading causes of death in BC patients with long-term survival rates [8]. A variety of studies have evaluated the risk factors for the occurrence of SPM, including age [6], different sites [9, 10], the expression of genes [11], the status of receptors [12, 13], and different therapeutic strategies, such as radiotherapy [6, 14] and chemotherapy [15]. However, few studies have illustrated the relationship between SPM and the survival of BC patients. A previous study reported that female BC patients with SPM tended to present poorer outcomes than those who only had BC in general [15]; however, the factors influencing the survival of BC patients with SPM remain to be clarified. Knowledge of the latency period, the time from the date of primary malignancy diagnosis to the SPM diagnosis, has been hypothesized to influence survival outcomes in malignant astrocytoma [16] and lung cancer [17]. In patients with malignant astrocytoma, those who developed an SPM at 11 months or later had better outcomes, and the risk of overall mortality decreased as the latency time increased [16]. Another study proposed that patients with primary lung cancer had a lower mortality when SPM was diagnosed earlier [17]. To our knowledge, there has been no discussion about the death risk of latency in patients with primary BC.

The nomogram has been widely used in the clinic as a prognostic prediction tool in oncology [18]. It can simplify the statistical predictive models in clinical decision making, by estimating the single numerical probability of an even, for instance death, for an individual patient [19]. Hence, the aim of this study was to screen independent factors associated with the survival of BC patients with SPM. Then, we predicted the survival probabilities of BC patients with SPM by establishing nomogram models. We believe the findings will guide individualized clinical management for professional and comprehensive treatment of BC patients with SPM.

## Materials and methods

### Data collection

Information on demographics, breast cancer diagnosis, second primary malignancy diagnosis, survival and other data were collected from 2002 to 2015 through a total of 50 hospitals in Shanghai, China. Shanghai is the largest metropolis with 16 districts in China and has witnessed the rapid development of China [20]. The tumour reporting system of the Cancer Registry in Shanghai was established in 2002; since then, the data of cancer patients have been utilized [21]. The date of diagnosis of breast cancer was set as the starting point of observation, and the end point was set on December 31, 2015. Patients received follow-up surveys every 6 months after providing informed consent to assess the survival status via home visit or telephone according to standard epidemiologic procedures [20, 22]. These patients were followed up until death or June 30, 2017, whichever came first. Data quality was checked and evaluated according to the criteria of the Chinese Cancer Registration [23] and IARC/IACR [23].

### Study participants

A total of 181 women with primary, nonmetastatic breast cancer and SPM were initially deemed eligible during 2002–2015 in Shanghai. Among these patients, 25 patients were diagnosed with first breast cancer before 2002 but were diagnosed with second primary malignancy between 2002 and 2015. Each of these participants was identified according to the International Classification of Diseases, 10th edition (ICD-10). We excluded 3 male breast cancer patients because of the low number of patients. Three patients who developed a third primary malignancy were excluded, which was beyond the scope of this study. Twelve patients were also excluded, as the IACR/IARC rules (accepted internationally) suggest that the time between the first and second primary malignancies should be more than 6 months [24, 25]. Finally, a total of 163 female breast cancer patients with a second primary malignancy were included in this study.

The quality assessment was conducted according to the applicable Enhancing the Quality and Transparency of Health Research (EQUATOR) reporting guidelines [26].

### Variables

The following demographic and clinical variables were analysed in our study: age, hospital grade, surgery, American Joint Committee on Cancer (AJCC) 6th Tumour node metastasis (TNM) staging classification [27], latency (the time interval between the date of primary breast cancer diagnosis and the date of second primary malignancy diagnosis [6]), and the sites of SPM.

### Statistical analysis

The primary outcomes of this study were overall survival (OS) and cancer-specific survival (CSS). OS was defined as death due to any cause, and CSS was defined as death due to breast cancer or SPM. The survival time was calculated in months from the date of BC diagnosis or SPM diagnosis to the endpoint (death or censoring). Chi-square or Fisher’s exact tests allowed for comparisons between categorical variables. The Kaplan–Meier method was used to compare survival curves with the log-rank test. Univariate and multivariate Cox regression analyses were performed to estimate the risk of survival with hazard ratios (HRs) and associated 95% confidence intervals (CIs). In all Cox regression models, the proportional hazards (PH) assumption was evaluated by a log-minus-log plot, hazard function plot and scaled and unscaled Schoenfeld residuals test. Variables that violated the PH [28] were transformed to a time-varying covariate with the time function and added to the Cox-PH model, and covariates were selected based on variables clinically important and with a *p* < 0.25 [29] in univariate analysis. To control the impact of confounders, we used the forward stepwise procedure based on the likelihood ratio test.

Two nomogram models were constructed to predict the OS of BC patients with SPM based on factors selected from multivariable Cox regression analysis with a *p* value less than 0.05. Model 1 was established to predict OS after BC diagnosis. Model 2 was established to predict OS after SPM diagnosis. A total of 163 patients were randomly divided into a training cohort and validation cohort, and each cohort included half of the patients [30]. The characteristics showed no significance between these two cohorts. Receiver characteristic curves (ROCs) and calibration curves were plotted and calculated to evaluate the calibration for predicting the survival rates of BC patients with SPM.

All statistical analyses and graphical representations were performed using GraphPad Prism (Version 8.0.1, Inc.), Statistical Package for the Social Sciences software version 26.0 (SPSS, Chicago, IL, USA), and R software 3.63 (R Foundation for Statistical Computing). All statistical tests were two-sided, and statistical significance was set at *p* < 0.05.

## Results

### Baseline characteristics

A total of 163 women breast cancer patients with SPM were enrolled in this study between 2002 and 2015. These patients were all over 24 years old. The baseline characteristics of the patients are presented in Table 1. The average age of the patients was 56.47 ± 11.62 years old. Among all 163 breast cancer patients, 77 (47.24%) developed SPM within 5 years, while 86 (52.76%) developed SPM beyond 5 years. As shown in Table 1, there were significant differences in surgery for breast cancer (*p* = 0.001) and SPM sites (*p* = 0.004). Compared to patients with a latency over 5 years, patients who had a latency less than 5 years received more surgery for breast cancer (61.04% vs. 38.37%, *p* = 0.001), and more SPMs occurred in the thyroid (28.57% vs. 6.98%,* p* = 0.004). All SPM sites are presented in Additional file 1. The number of patients with SPM in the colon and rectum ranked first (n = 30), followed by the thyroid (n = 28) and lung and bronchus (n = 24), and each other site included fewer than 10 patients. Thus, the SPM sites in the colon and rectum, thyroid and lung and bronchus were included in further analysis.

**Table 1:** Demographics and characteristics of 163 breast cancer patients with a second primary malignancy, n (%)

Variable	Total number (N = 163)	Latency (years)		*p*
		≤ 5 (n = 77)	> 5 (n = 86)	
Age of BC diagnosis (years)	56.47 ± 11.62^a^			
< 56	102 (62.58)	50 (64.94)	52 (60.47)	0.556
≥ 56	61 (37.42)	27 (35.06)	34 (39.53)	
Age of SPM diagnosis (years)	62.42 ± 11.99^a^			0.418
< 62 (ref)	94 (57.67)	63 (60.00)	31 (53.45)	
≥ 62	69 (42.33)	42 (40.00)	27 (46.55)	
TNM stage of BC				0.123
I + II	119 (73.01)	62 (80.52)	57 (66.28)	
III + IV	12 (7.36)	4 (5.19)	8 (9.30)	
Unclassified	32 (19.63)	11 (14.29)	21 (24.42)	
TNM stage of SPM				0.161
I + II	59 (36.20)	33 (42.90)	26 (30.23)	
III + IV	36 (22.10)	13 (16.88)	23 (26.74)	
Unclassified	68 (41.72)	31 (40.26)	37 (43.02)	
BC therapeutic hospital grade				
Secondary	62 (38.04)	30 (38.96)	32 (37.21)	0.818
Tertiary	101 (61.96)	47 (61.04)	54 (62.79)	
SPM therapeutic hospital grade				
Secondary	38 (23.31)	16 (20.78)	22 (25.58)	0.469
Tertiary	125 (76.69)	61 (79.22)	64 (74.42)	
Surgery for BC				0.001
Yes	80 (49.07)	47 (61.04)	33 (38.37)	
No	58 (35.58)	26 (33.76)	32 (37.21)	
Unknown	25 (15.34)	4 (5.19)	21 (24.42)	
Surgery for SPM				0.653
Yes	43 (55.84)	45 (52.33)	88 (53.99)	
No	75 (46.01)	34 (44.16)	41 (47.67)	
SPM sites				0.004
Colon and rectum	30 (18.40)	12 (15.58)	18 (20.93)	
Thyroid	28 (17.18)	22 (28.57)	6 (6.98)	
Lung and bronchus	24 (14.72)	9 (11.69)	15 (17.44)	
Others	81 (49.69)	34 (44.16)	47 (54.65)	
Total number of patients	163 (100.00)	77 (47.24)	86 (52.76)	

*BC* breast cancer, *TNM* tumour node metastasis, *SPM* second primary malignancy

^a^Mean ± SD

Altogether, 45 (27.61%) deaths occurred among 163 patients during the 101.5-months median follow-up. At the end of follow-up, 74 patients had died, among whom 73 patients had died from cancer and only one patient had died from other causes. All patients survived at the 1-year follow-up, and the 5-years, 10-years and 15-years OS rates were 88.34%, 64.42% and 56.44%, respectively (Table 2).

**Table 2:** Overall survival rate of breast cancer patients who developed a second primary malignancy with different characteristics, n (%)

Variables	1-year			3-years			5-years			10-years			15-years		
	Survival	Death	*p*	Survival	Death	*p*	Survival	Death	*p*	Survival	Death	*p*	Survival	Death	*p*
Number	163 (100.00)	0		153 (89.57)	10 (6.13)		144 (88.34)	19 (11.66)		105 (64.42)	58 (35.58)		92 (56.44)	71 (43.56)	
Age at BC diagnosis (years)		–			1.000			0.145			0.147			**0.015**	
< 56	102 (100.00)	0		96 (94.12)	6 (5.88)		93 (91.18)	9 (8.82)		70 (68.63)	32 (31.37)		65 (63.73)	37 (36.28)	
≥ 56	61 (100.00)	0		57 (93.44)	4 (6.56)		51 (83.61)	10 (16.39)		35 (57.38)	26 (42.62)		27 (44.26)	34 (55.74)	
Age at SPM diagnosis (years)		–			1.000			0.983			0.418			**0**.**011**	
< 62	94 (100.00)	0		88 (93.62)	6 (6.38)		83 (88.30)	11 (11.70)		63 (67.02)	31 (32.98)		61 (64.89)	33 (35.11)	
≥ 62	69 (100.00)	0		65 (94.20)	4 (5.80)		61 (88.41)	8 (11.59)		42 (60.87)	27 (39.13)		31 (44.93)	38 (55.07)	
TNM stage of BC^a^			–			0.229			0.975			0.810			0.252
I + II	119 (100.00)	0		111 (93.28)	8 (6.72)		105 (88.24)	14 (11.76)		79 (66.39)	40 (33.61)		70 (58.82)	49 (41.18)	
III + IV	12 (100.00)	0		10 (83.33)	2 (16.67)		10 (83.33)	2 (16.67)		7 (58.33)	5 (41.67)		5 (41.67)	7 (58.33)	
TNM stage of SPM^a^			–			1.000			0.135			** < 0.001**			** < 0.001**
I + II	59 (100.00)	0		57 (96.61)	2 (3.39)		57 (96.61)	2 (3.39)		52 (88.14)	7 (11.86)		50 (84.74)	9 (15.25)	
III + IV	36 (100.00)	0		35 (97.22)	1 (2.78)		31 (86.11)	5 (13.89)		16 (44.44)	20 (55.56)		13 (36.11)	23 (63.89)	
BC therapeutic hospital grade		–			0.838			0.537			0.302			0.103	
Tertiary	101 (100.00)	0		94 (93.07)	7 (6.93)		88 (87.13)	13 (12.87)		62 (61.39)	39 (38.61)		52 (51.49)	49 (48.51)	
Secondary	62 (100.00)	0		59 (95.16)	3 (4.84)		56 (90.32)	6 (9.68)		43 (69.35)	19 (30.65)		40 (64.52)	22 (35.48)	
SPM therapeutic hospital grade		–			0.896			0.537			0.338			**0.042**	
Tertiary	125 (100.00)	0		118 (94.40)	7 (5.60)		112 (89.60)	13 (10.40)		83 (66.40)	42 (33.60)		76 (60.80)	49 (39.20)	
Secondary	38 (100.00)	0		35 (92.11)	3 (7.89)		32 (84.21)	6 (15.79)		22 (57.89)	16 (42.11)		16 (42.11)	22 (57.89)	
Surgery for BC^b^			–			1.000			0.269			0.761			0.859
Yes	80 (100.00)	0		83 (94.32)	5 (5.68)		80 (90.91)	8 (9.09)		49 (61.25)	31 (38.75)		46 (57.50)	34 (42.50)	
No	58 (100.00)	0		70 (93.33)	5 (6.67)		64 (85.33)	11 (14.67)		37 (63.79)	21 (36.21)		34 (58.62)	24 (41.38)	
Surgery for SPM			–			0.102			0.192			** < 0.001**			** < 0.001**
Yes	88 (100.00)	0		82 (93.18)	6 (6.82)		77 (87.50)	11 (12.50)		68 (77.27)	20 (22.73)		62 (70.45)	26 (29.55)	
No	75 (100.00)	0		64 (85.33)	11 (14.67)		60 (80.00)	15 (20.00)		37 (49.33)	38 (50.67)		30 (40.00)	45 (60.00)	
SPM sites^c^															
Colon and rectum			–			0.259			0.530			0.259			0.211
Yes	30 (100.00)	0		30 (100.00)	0 (0)		28 (93.33)	2 (6.67)		22 (73.33)	8 (26.67)		20 (66.67)	10 (33.33)	
No	133 (100.00)	0		123 (92.48)	10 (7.52)		116 (87.22)	17 (12.78)		83 (62.41)	50 (37.59)		72 (54.14)	61 (45.86)	
Thyroid			–			0.292			0.254			** < 0.001**			** < 0.001**
Yes	28 (100.00)	0		28 (100.00)	0		27 (96.43)	1 (3.57)		27 (96.43)	1 (3.57)		27 (96.43)	1 (3.57)	
No	135 (100.00)	0		125 (92.59)	10 (7.41)		117 (86.67)	18 (13.33)		78 (57.78)	57 (42.22)		65 (48.15)	70 (51.85)	
Lung and bronchus		–			0.344			0.628			0.832			0.491	
Yes	24 (100.00)	0		21 (87.50)	3 (12.50)		20 (83.33)	4 (16.67)		15 (62.50)	9 (37.50)		12 (50.00)	12 (50.00)	
No	139 (100.00)	0		132 (94.96)	7 (5.04)		124 (89.21)	15 (10.79)		90 (64.75)	49 (35.25)		80 (57.55)	59 (42.45)	
Latency (years)			–			**0.002**			** < 0.001**			0.647			**0.017**
≤ 5	77 (94.81)	0		67 (87.01)	10 (12.99)		58 (75.32)	19 (24.68)		51 (66.23)	26 (33.77)		51 (66.23)	26 (33.77)	
> 5	86 (100.00)	0		86 (100.00)	0 (0)		86 (100.00)	0 (0)		54 (62.79)	32 (37.21)		41 (47.67)	45 (52.33)	

Bold means statistic significance

*BC* breast cancer, *TNM* tumour node metastasis, *SPM* second primary malignancy

^a^Patients with an unclassified TNM stage were not included

^b^Patients with an unknown status of surgery were not included

^c^The number of SPM sites in colon and rectum, thyroid and lung and bronchus ranked as the top three, were included

### Univariate analysis of factors associated with poor outcomes of breast cancer patients with second primary malignancy

**Fig. 1:**
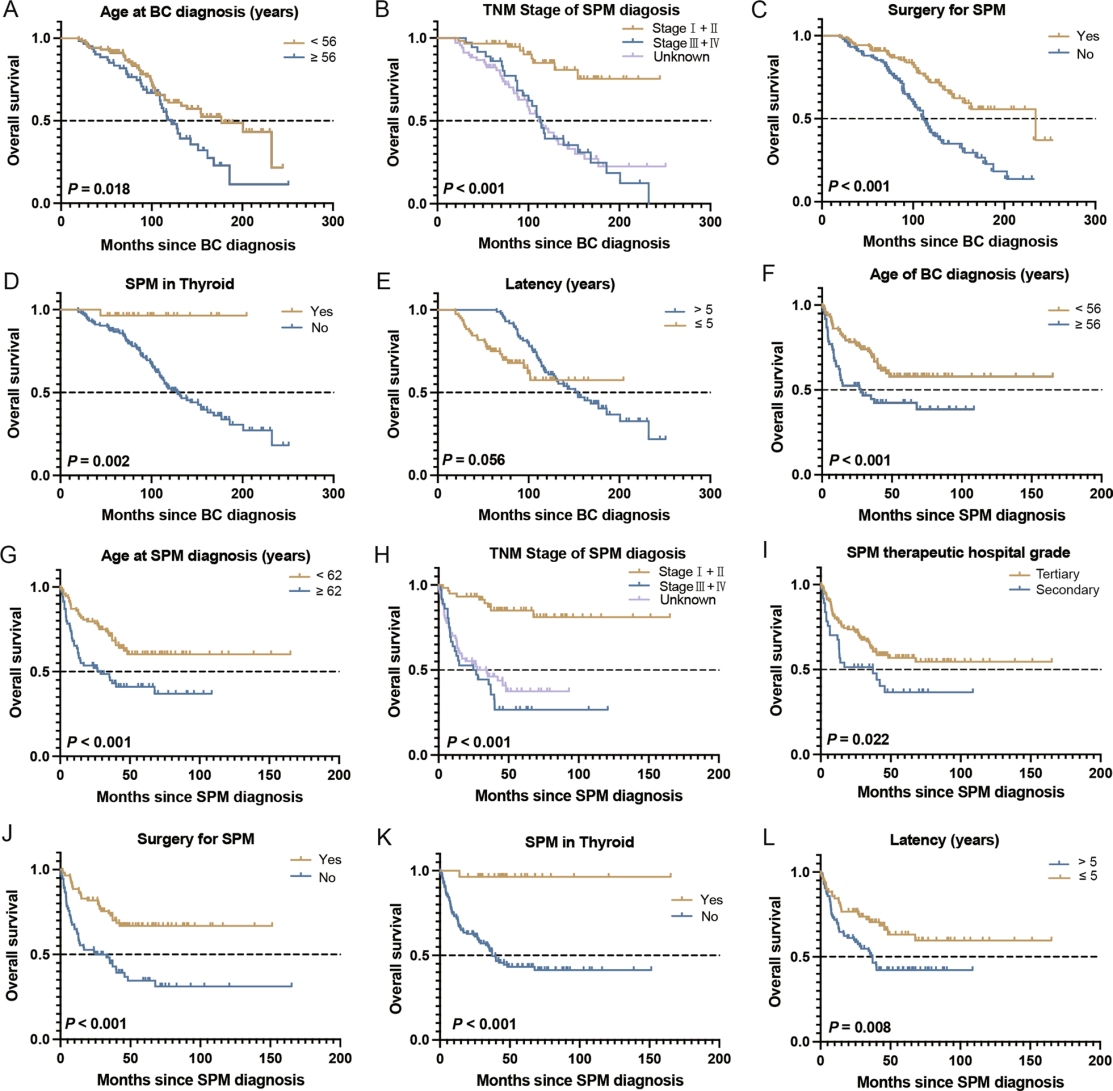
Overall survival of breast cancer patients with SPM with different characteristics. **A**–**E** Survival from BC diagnosis. **F**–**L** Survival from SPM diagnosis. Overall survival with different characteristics in (**A**, **F**) age at BC diagnosis, **B**, **H** TNM stage of SPM, (**C**, **J**) surgery for SPM, **D**, **K** SPM in thyroid, **E**, **L** latency, **G** age at SPM diagnosis, **I** SPM therapeutic hospital grade. BC, breast cancer; SPM, second primary malignancy

TO screen the risk factors for overall and cancer-specific survival from BC and SPM, we conducted univariate analysis of all variables. As shown in Table 3, the HRs of variables were similar to the OS and CSS both from BC and SPM diagnosis, as there was only one patient who died from noncancer causes. For survival from BC, factors of older age (≥ 56 years old), late TNM stage of SPM (III + IV), nonsurgery for SPM, and SPM occurring in nonthyroidsites were significantly associated with poor survival (*p* < 0.05). The latency was transformed to a time-varying covariate with the time function. The hazard ratio (HR) for breast cancer patients with a longer latency (> 5 years) was 0.002 (95% CI 0–0.07, *p* = 0.001) for both OS and CSS, and the interaction between latency and time had an HR of 1.08 (*p* = 0.001). For survival from SPM, factors of older age at BC (≥ 56 years old) and SPM (≥ 62 years old) diagnosis, late TNM stage of SPM (III + IV), SPM treated in secondary hospital, nonsurgery for SPM, SPM occurring in nonthyroid sites and latency were significantly associated with poor survival (*p* < 0.05). These significant risk factors for OS were also analysed in Kaplan‒Meier curves, as shown in Fig. 1.

**Table 3:** Univariate analysis of factors influencing the survival time of breast cancer patients with second primary malignancy

Variables	OS^a^		CSS^a^		OS: SPM^b^		CSS: SPM^b^	
	HR (95% CI)	*p*	HR (95% CI)	*p*	HR (95% CI)	*p*	HR (95% CI)	*p*
*Age at BC diagnosis (years)*								
≥ 56 versus < 56	1.68 (1.06–2.67)	**0.027**	1.64 (1.03–2.60)	**0.038**	2.00 (1.26–3.16)	**0.003**	1.95 (1.22–3.10)	**0.005**
*Age at SPM diagnosis (years)*								
≥ 62 versus < 62	1.43 (0.90–2.27)	0.126	1.40 (0.88–2.22)	0.156	2.15 (1.35–3.42)	**0.001**	2.10 (1.32–3.35)	**0.002**
*TNM stage of BC*								
I + II (ref)								
III + IV	1.29 (0.58–2.85)	0.530	1.32 (0.60–2.92)	0.498	1.57 (0.71–3.47)	0.261	1.61 (0.73–3.55)	0.241
Unclassified	1.05 (0.60–1.84)	0.869	1.07 (0.61–1.88)	0.816	1.24 (0.71–2.19)	0.445	1.27 (0.72–2.24)	0.404
*TNM stage of SPM*								
I + II (ref)								
III + IV	5.08 (2.38–10.84)	** < 0.001**	5.07 (2.38–10.83)	** < 0.001**	7.13 (3.33–15.28)	** < 0.001**	7.17 (3.34–15.36)	** < 0.001**
Unclassified	5.03 (2.44–10.40)	** < 0.001**	4.90 (2.37–10.14)	** < 0.001**	5.65 (2.72–11.74)	** < 0.001**	5.53 (2.66–11.50)	** < 0.001**
*BC therapeutic hospital grade*								
Secondary versus tertiary	0.82 (0.50–1.33)	0.412	0.78 (0.48–1.28)	0.328	0.72 (0.44–1.18)	0.195	0.69 (0.42–1.14)	0.146
*SPM therapeutic hospital grade*								
Secondary versus tertiary	1.40 (0.86–2.29)	0.181	1.34 (0.81–2.21)	0.251	1.78 (1.08–2.93)	**0.024**	1.70 (1.02–2.83)	**0.040**
*Surgery for BC*								
No (ref)								
Yes	1.24 (0.74–2.10)	0.419	1.29 (0.76–2.20)	0.340	1.02 (0.61–1.73)	0.929	1.07 (0.63–1.82)	0.806
Unknown	0.68 (0.34–1.37)	0.280	0.71 (0.35–1.43)	0.337	1.64 (0.87–3.09)	0.127	1.71 (0.90–3.24)	0.100
*Surgery for SPM*								
Yes versus No	0.40 (0.25–0.65)	** < 0.001**	0.41 (0.25–0.66)	** < 0.001**	0.36 (0.22–0.57)	** < 0.001**	0.36 (0.22–0.59)	** < 0.001**
*SPM sites*								
Colon and rectum								
No versus yes	1.77 (0.91–3.45)	0.094	1.74 (0.89–3.40)	0.104	1.52 (0.80–2.88)	0.202	1.50 (0.79–2.84)	0.219
Thyroid								
No versus yes	11.92 (1.66–85.84)	**0.014**	11.77 (1.63–84.80)	**0.014**	20.65 (2.87–148.69)	**0.003**	20.44 (2.84–147.20)	**0.003**
Lung and bronchus								
No versus yes	0.90 (0.49–1.64)	0.723	0.88 (0.48–1.61)	0.686	0.70 (0.38–1.27)	0.239	0.68 (0.38–1.25)	0.216
*Latency (years)*								
> 5 versus ≤ 5	0.002 (0–0.07)	**0.001**	0.002 (0–0.07)	**0.001**	1.89 (1.17–3.05)	**0.009**	1.85 (1.14–3.00)	**0.012**
*Latency* × *Time*^*c*^	1.08 (1.03–1.13)	**0.001**	1.08 (1.03–1.12)	**0.001**	–	–	–	–

Bold means statistic significance

*BC* breast cancer, *TNM* tumour node metastasis, *SPM* second primary malignancy

^a^Survival time from BC diagnosis

^b^Survival time from SPM diagnosis

^c^Latency was transformed into a time-varying covariate to assess the survival time from BC diagnosis

### Multivariate analysis of factors influencing the survival time of breast cancer patients with a second primary malignancy

To determine which factors were independently associated with the overall survival of breast cancer patients with SPM, multivariate Cox regression analysis was applied. The age of BC and SPM diagnosis, TNM stage of SPM, SPM therapeutic hospital grade, surgery for SPM, sites of SPM and latency were selected for models. The final models are shown in Table 4. The independent factors of TNM stage of SPM, surgery for SPM, SPM in colon and rectum and thyroid remained significant in the forward stepwise multivariable model for predicting overall survival from BC and SPM diagnosis. Notably, the late TNM stage of SPM (III + IV) was an independent factor for predicting poor survival from BC (HR = 4.68, 95% CI 2.14–10.25,* p* < 0.001) and SPM (HR = 5.76, 95% CI 2.65–12.51,* p* < 0.001). In addition, surgery for SPM, SPM in the colon and rectum or thyroid was a protective factor for survival from BC (HR 0.60, 0.49 and 0.08, respectively;* p* < 0.05) and SPM (HR 0.58, 0.43 and 0.07, respectively;* p* < 0.05). In the Cox model with time-varying covariates for survival from BC diagnosis, the longer latency (> 5 years) had an HR of 0.002 (*p* < 0.001), and the interaction between latency and time had an HR of 1.07 (*p* = 0.003). In the model for survival from SPM diagnosis, an older age of SPM diagnosis (≥ 56 years) was associated with poor survival after SPM diagnosis (HR = 1.80, 95% CI 1.10–2.83,* p* = 0.019).

**Table 4:** Factors influencing the overall survival of breast cancer patients with second primary malignancy by multivariate Cox regression model

Variables	OS^a^			OS: SPM^b^		
	B	HR (95% CI)	*p*	B	HR (95% CI)	*p*
*Age at BC diagnosis (years)*						
≥ 56 versus < 56	–^c^	–^c^	0.324	0.57	1.80 (1.10–2.83)	**0.019**
*TNM stage of SPM*						
I + II (ref)						
III + IV	1.54	4.68 (2.14–10.25)	** < 0.001**	1.80	6.05 (2.76–13.26)	** < 0.001**
Unclassified	1.47	4.34 (2.06–9.16)	** < 0.001**	1.55	4.71 (2.25–9.89)	** < 0.001**
*Surgery for SPM*						
Yes versus No	− 0.51	0.60 (0.36–1.00)	**0.048**	− 0.55	0.58 (0.35–0.95)	**0.029**
*SPM sites*						
Other sites (ref)						
Colon and rectum	− 0.72	0.49 (0.25–0.98)	**0.043**	− 0.84	0.43 (0.22–0.83)	**0.012**
Thyroid	− 2.50	0.08 (0.01–0.61)	**0.014**	− 2.63	0.07 (0.01–0.53)	**0.010**
*Latency (years)*						
> 5 versus ≤ 5	− 6.47	0.002 (0–0.05)	** < 0.001**	–^c^	–^c^	0.225
*Latency* × *Time*^*d*^	0.07	1.07 (1.02–1.12)	**0.003**	–	–	–

Bold means statistic significance

*BC* breast cancer, *TNM* tumour node metastasis, *SPM* second primary malignancy

^a^Survival time from BC diagnosis

^b^Survival time from SPM diagnosis

^c^Coefficient and HR estimates were not available because the variable was excluded from the model

^d^Latency was transformed into a time-varying covariate to assess the survival time from BC diagnosis

### Nomogram establishment and validation

**Fig. 2:**
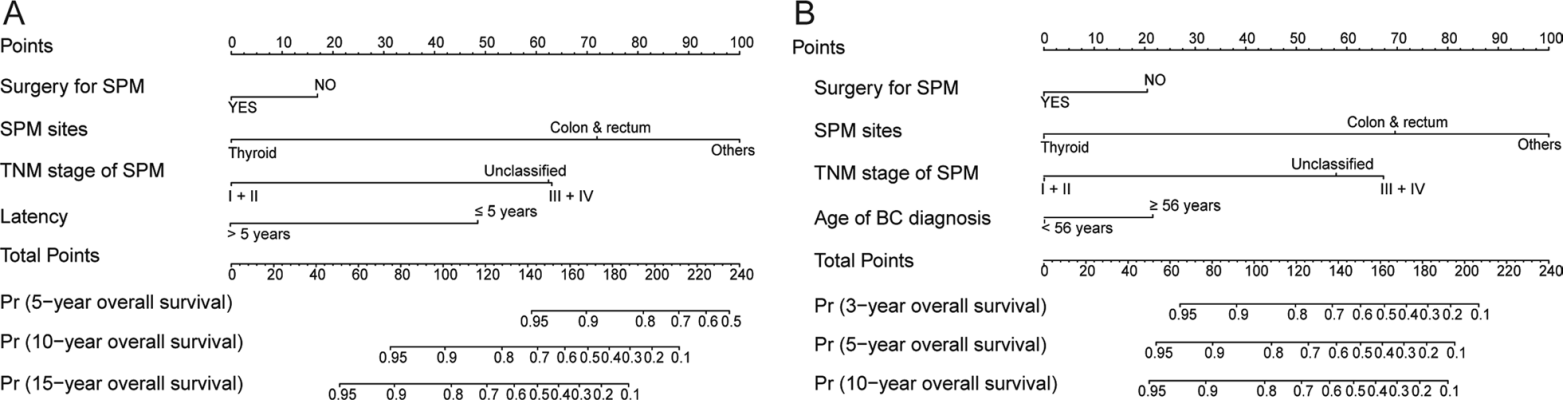
Nomograms for overall survival prediction of female BC patients with SPM. **A** Model 1: nomogram for predicting survival probability after BC diagnosis. The points according to the presence/absence of the categorized variables could be obtained according to the point of the horizontal axes. According to the sum of total points, a vertical line was drawn through the total point axis and survival axes to determine the probabilities of 5-, 10- and 15-years OS. **B** Model 2: nomogram for predicting 3-, 5- and 10-years OS after SPM diagnosis. BC, breast cancer; TNM, tumour node metastasis; SPM, second primary malignancy; OS, overall survival

To establish predictive nomogram models for the survival of BC patients with SPM, we selected the independent factors from multivariate Cox analysis and found no significant collinearity. A total of 163 patients were randomly divided into a training cohort and validation cohort with no significant differences in characteristics (see Additional file 2). Model 1 included possible predictors of TNM stage of SPM, surgery for SPM, sites of SPM and latency to predict OS probabilities after BC diagnosis (Fig. 2A). Model 2 included possible predictors of age at BC diagnosis, TNM stage of SPM, surgery for SPM and sites of SPM to predict OS probabilities after SPM diagnosis (Fig. 2B). For an individual patient, the total nomogram-related score was calculated by summing the points corresponding to the status of the predictors.

**Fig. 3:**
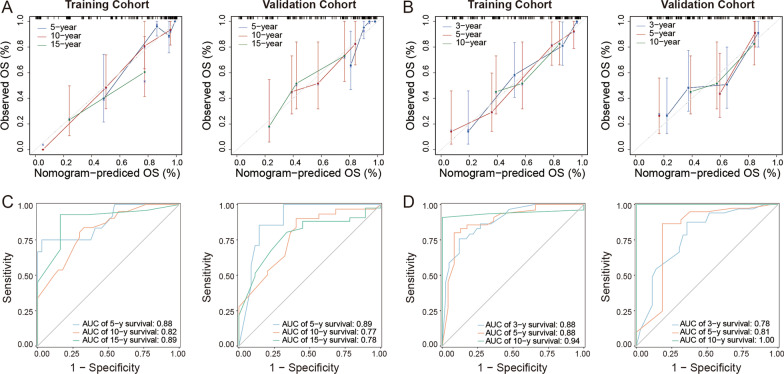
Validation of two nomograms for overall survival prediction of female BC patients with SPM. **A** Calibration and **C** ROC curves of 5-, 10- and 15-years OS from BC diagnosis in the training cohort (left) and validation cohort (right). The grey line represents the performance of the ideal nomogram where the predicted probability perfectly corresponds to the observed probability. **B** Calibration and **D** ROC curves of 3-, 5- and 10-years OS from SPM diagnosis in the training cohort (left) and validation cohort (right). ROC, receiver operating characteristic; BC, breast cancer; SPM, second primary malignancy; OS, overall survival

The calibration curves showed that the nomograms had relatively good consistency between the predicted and observed survival probabilities in the training and validation cohorts in both models (Fig. 3A, B). The ROC curves presented better discriminatory capacity of the nomogram models. In model 1 (Fig. 3C), the areas under the curve (AUCs) were 0.88, 0.82 and 0.89 in the training cohort and 0.89, 0.77 and 0.78 in the validation cohort for 5-, 10- and 15-years OS prediction, respectively. In model 2 (Fig. 3D), the AUCs were 0.88, 0.88 and 0.94 in the training cohort and 0.78, 0.81 and 1.00 in the validation cohort for 3-, 5- and 10-years OS prediction, respectively.

## Discussion

Previous studies have claimed that SPM is one of the leading causes of death in BC patients. Our study found that the survival rate of female BC patients with SPM was lower than that of patients with only BC, which proved the threat of SPM to survival. Furthermore, factors that affected the OS of femaleBC patients with SPM were SPM, including TNM stage, surgery, sites, and latency. In addition, younger age at BC diagnosis predicted poor survival in these participants. Based on these risk factors, two nomograms established to predict survival probabilities after BC and SPM were diagnosed presented better discriminatory capacity, which demonstrated potential predictive value for BC management in the clinic.

Among the patients in our study, those who developed SPM had a survival rate of 75.32%, while those who did not develop SPM had a survival rate of 100% at the 5-years follow-up. In addition, the 10-years and 15-years survival rates of BC patients who developed SPM were decreased to 64.42% and 56.44%, respectively. It has been proven that the survival rate of BC is associated with socioeconomic status and culture [31]. In America, a report based on the Surveillance, Epidemiology, and End Results (SEER) database showed that the 5-years survival rate of female BC patients was 91.1% during 2008–2014 [32]. In China, the 5-years female BC survival rate was 80.4–83.3% from 2005 to 2014, which was relatively lower than that in the US [4]. In our study, the 5-years survival rate of female BC patients was 88.34% during 2002–2015, which is higher than the reported 80.4–83.3%. This discrepancy may be due to the economic advantages and advanced medical care available in Shanghai, but this needs to be further clarified.

In addition, regarding the age of BC patients, a previous study based on SEER found that in the US, 60.3% of female BC patients with SPM were 60 years old and older [6]. In our study, these patients (< 56 years old) accounted for 62.58%, which indicated that BC patients with SPM tend to be younger in China. Interestingly, it is a protective factor compared to those who were younger than 56 years old when predicting the overall survival probability of BC patients after developing SPM.

In this study, we constructed univariable and multivariable analyses as well as well-discriminatory prognostic nomogram models to predict OS in BC patients with SPM. TNM stage is an important predictor of cancer outcomes. Consistent with prior studies in other cancers [33–35], we found that late-stage (III + IV) SPM was associated with poor survival in BC patients with SPM. One reason for this result may be a delayed diagnosis of SPM in that patients diagnosed with late-stage SPM may not receive early detection and comprehensive treatment in a timely manner [36]. Evidence-based studies need to be further analysed. This is a reminder that early cancer screening is of importance even for a patient already diagnosed with BC.

Surgery is a vital treatment for cancer therapies. Although a study pointed out that women nursing home residents who received BC surgery had high 1-year mortality [37], our study showed that surgical treatment for primary BC had no significant effect on the OS or CSS for BC patients with SPM. However, receiving surgical treatment for SPM was shown to be protective for survival, which is similar to the findings from a study on nasopharyngeal tumours [38]. Our results may provide a reference for clinicians making treatment decisions for BC patients with SPM.

Different SPM sites were related to significant survival outcomes. In our results, SPM in the colon and rectum and thyroid predicted better survival. Colorectal cancer (CRC) is the third most common cancer worldwide [39] and the top five most common cancers in China [40]. It has been proven that the CRC screening program could improve CRC patient survival in developed and developing countries [41], which provides a reason for our result. In particular, BC patients with SPM in the thyroid had an excellent 15-years OS of 96.43%. Although the incidence of thyroid cancer has increased recently worldwide, thyroid cancer patients still have better survival rates; for instance, the 5-years survival rate of thyroid cancer patients reached 98% in America [42]. It is worth noting that a high cumulative radioiodine dose increased the incidence of SPM in thyroid cancer patients, which is a vital risk factor for death [43]. Therefore, stricter surveillance of BC patients is needed to facilitate early diagnosis and screening of SPM, and more professional and comprehensive treatment should be offered.

The risk effect of latency period on developing SPM has been studied in many cancers, but the association between latency and survival has rarely been studied. Previous studies proved that longer latency was associated with lower mortality in astrocytoma [16] and lung cancer [17]. In BC, our study first pointed out that developing an SPM with a longer latency (more than 5 years) was associated with a decreased risk of all-cause death, which is consistent with related studies of other cancers. A literature showed that adolescents and young adults who developed BC within 5 years had a 2.6-fold increased risk of death [7], however, BC was an SPM rather than a primary cancer in this study.

In the present study, two nomograms were established based on surgical treatment for SPM, SPM sites, TNM stage of SPM, latency, and age of BC diagnosis to facilitate a quantitative assessment of survival of BC patients who developed SPM once their clinical data were available. Internal validation statistically indicated that the current nomograms were accurate in their predictive abilities. Particularly, in predicting BC patients 10-years OS from SPM diagnosis, the AUC reached 0.94 and 1.00 in the training and validation cohorts, respectively. These models would enable a survival prediction of BC patients who developed an SPM, but TNM stage could not be classified in the clinic. Since the factors of latency and surgery were included, the nomogram could longitudinally evaluate overall survival and weight the risk of various management options, including surgery, at optimal time points.

Few studies have illustrated the factors influencing the survival of BC patients with SPM. Our study first identified latency, which affected the overall survival of these patients. It is worth paying attention to latency because longer latency is associated with improved survival. In addition, with respect to exploring survival risk factors for BC patients with SPM, obesity and metabolic diseases should be considered and are worthy of study, as they are important risk factors for cancers including BC [44, 45].

There are several limitations in the present study. First, although our data were collected from 50 hospitals in Shanghai during 2002–2015, the study only included 163 BC female patients with SPM, which was relatively small and affected the calibration of the nomogram models. Second, due to the long-term follow-up, some patient information about surgery for BC was not completed, which resulted in some missing bias. Third, we did not obtain health data related to obesity and metabolic diseases of participants, which may be risk factors for breast cancer [45]. Finally, we evaluated the nomogram models by internal validation, and they need to be externally validated in future real-world studies to assess the accuracy and verify their utility for clinicians.

## Conclusion

IN summary, for BC patients with SPM, we determined the SPM factors for TNM stage, surgery, and sites as independent predictors for OS from BC diagnosis and SPM diagnosis. The latency might be used in predicting 5-, 10- and 15-years survival from BC diagnosis. In addition, the age of BC diagnosis might be used in predicting 3-, 5- and 10-years survival from SPM diagnosis. The nomograms may provide a reference for individualized survival prediction and management options for BC patients with SPM.

## Supplementary Information

**Additional file1**. **Figure S1**: The sites of second primary malignancy in female breast cancer patients.

**Additional file2**. **Table S1**: Clinical characteristics in the training cohort and validation cohort.

## Data Availability

The data presented in this study are available upon request from the corresponding author. The data are not publicly available due to privacy and ethical restrictions.

## Abbreviations

BC: Breast cancer
SPM: Second primary malignancy
ICD-10: International Classification of Diseases, 10th edition
AJCC: American Joint Committee on Cancer
TNM: Tumour node metastasis
OS: Overall survival
CSS: Cancer-specific survival
HRs: Hazard ratios
CIs: Confidence intervals
PH: Proportional hazard
ROCs: Receiver characteristic curves
