# Supplementary figures and images for: Predictive models for overall survival in breast cancer patients with a second primary malignancy: a real-world study in Shanghai, China
Source: BMC Womens Health. 2022 Dec 6;22:498. doi: 10.1186/s12905-022-02079-0 (PMC9724326; doi:10.1186/s12905-022-02079-0)

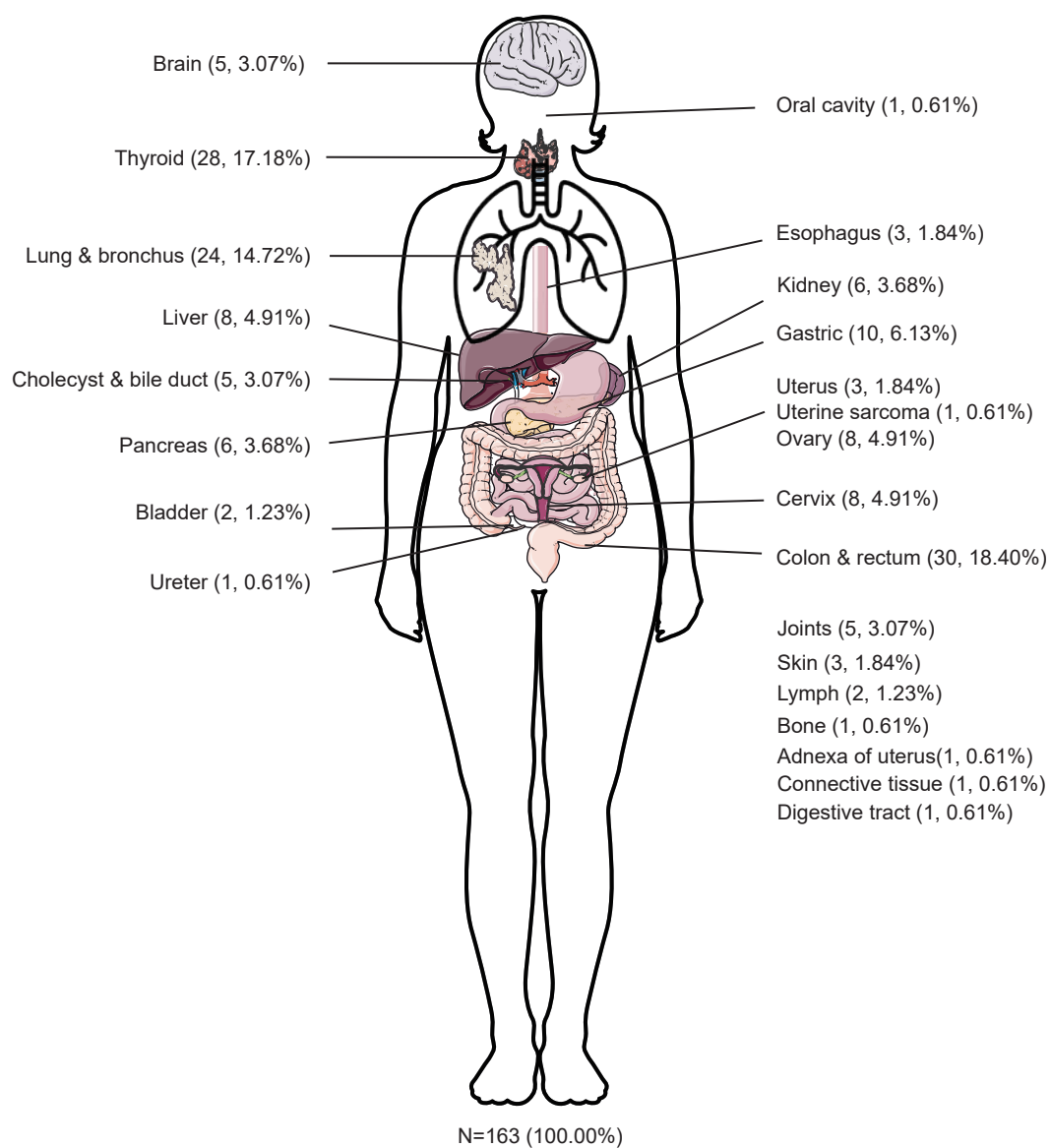

Figure S1. The sites of second primary malignancy in female breast cancer patients.

Supplement: Supplementary file 1 — Additional file1. Figure S1: The sites of second primary malignancy in female breast cancer patients. [file 12905_2022_2079_MOESM1_ESM.pdf]
