# Supplementary material for: Predictive models for overall survival in breast cancer patients with a second primary malignancy: a real-world study in Shanghai, China
Source: BMC Womens Health. 2022 Dec 6;22:498. doi: 10.1186/s12905-022-02079-0 (PMC9724326; doi:10.1186/s12905-022-02079-0)
Supplement: Supplementary file 2 — Additional file2. Table S1: Clinical characteristics in the training cohort and validation cohort. [file 12905_2022_2079_MOESM2_ESM.docx]

# Table S1. Clinical characteristics in the training cohort and validation cohort.

| **Variable** | **Training cohort**  **n (%)** | **Validation cohort**  **n (%)** | ***P*** |
| --- | --- | --- | --- |
| **Age of BC diagnosis (years)** |  |  | **0.919** |
| < 56 | 51 (62.20) | 51 (62.96) |  |
| ≥ 56 | 31 (37.80) | 30 (37.04) |  |
| **Age of SPC diagnosis (years)** |  |  | **0.587** |
| < 62 | 49 (59.76) | 45 (55.56) |  |
| ≥ 62 | 33 (40.24) | 36 (44.44) |  |
| **TNM stage of BC** |  |  | **0.174** |
| I+II | 59 (71.95) | 60 (74.07) |  |
| III+IV | 9 (10.98) | 3 (3.70) |  |
| Unclassified | 14 (17.07) | 18 (22.22) |  |
| **TNM stage of SPM** |  |  | **0.068** |
| I+II | 35 (42.68) | 24 (29.63) |  |
| III+IV | 20 (24.39) | 16 (19.75) |  |
| Unclassified | 27 (32.93) | 41 (50.62) |  |
| **BC therapeutic hospital grade** |  |  | **0.701** |
| Secondary | 52 (63.41) | 49 (60.49) |  |
| Tertiary | 30 (36.59) | 32 (39.51) |  |
| **SPM therapeutic hospital grade** |  |  | **0.248** |
| Secondary | 66(80.49) | 59 (72.84) |  |
| Tertiary | 16 (19.51) | 22 (27.16) |  |
| **Surgery for BC** |  |  | **0.283** |
| No | 34 (41.46) | 24 (29.63) |  |
| Yes | 37 (45.12) | 43 (53.09) |  |
| Unknown | 11 (13.41) | 14 (17.28) |  |
| **Surgery for SPM** |  |  | **0.476** |
| No | 40 (48.78) | 35 (43.21) |  |
| Yes | 42 (51.22) | 46 (56.79) |  |
| **Sites of SPM** |  |  | **0.328** |
| Colon & rectum | 15 (18.29) | 15 (18.52) |  |
| Thyroid | 18 (21.95) | 10 (12.35) |  |
| Lung & bronchus | 13 (15.85) | 11 (13.58) |  |
| Others | 36 (43.90) | 45 (55.55) |  |
| **Total number of patients** | 82 | 81 |  |

BC, breast cancer; TNM, tumour node metastasis; SPM, second primary malignancy
